# MAMA—Mandibular Advancement Magnetic Appliance: A Digital Workflow and a CAD–CAM Development of a New Mandibular Advancement Device for the Treatment of Obstructive Sleep Apnea Syndrome

**DOI:** 10.3390/dj13030104

**Published:** 2025-02-27

**Authors:** Riccardo Nucera, Enrico Nastro Siniscalchi, Giancarlo Consolo, Luigi Calabrese, Daniela Caccamo, Angela Mirea Bellocchio, Marco Portelli

**Affiliations:** 1 Department of Biomedical, Dental Science and Morphological and Functional Images, University of Messina, 98100 Messina, Italy; riccardo.nucera@unime.it (R.N.); enrico.nastrosiniscalchi@unikore.it (E.N.S.); daniela.caccamo@unime.it (D.C.); angelamirea@live.it (A.M.B.); 2 Department of Mathematical and Computer Sciences, Physical Sciences and Earth Sciences, University of Messina, 98100 Messina, Italy; giancarlo.consolo@unime.it; 3 Department of Engineering, University of Messina, 98100 Messina, Italy; lcalabrese@unime.it

**Keywords:** OSAS, mandibular advancement device, rare-earth magnets

## Abstract

**Background/Objectives:** Mandibular advancing devices (MADs) are removable intraoral apparatuses to use during sleep that modify the spatial position of the mandible, increasing airway patency and improving respiratory function at night in patients with obstructive sleep apnea syndrome (OSAS). **Methods:** In this work, a new mandibular advancement device useful for mild-to-moderate OSAS patients is presented. It is developed through a CAD–CAM process and involves a passive propulsion of the mandible thanks to the attraction of rare-earth magnets positioned in the thickness of two thermally molded PET-G devices. The use of a PET-G device compared to traditional resin ones offers several clinical advantages related to the innovative characteristics of this polymer, which allows the fabrication of thinner devices, with high resistance to fluid corrosion, resulting in less bulk inside the oral cavity. **Results:** The innovative feature of the device proposed by the authors is that mandibular propulsion induced by the attraction of the magnetic jigs is not affected by a patient’s mandibular posture during sleep. **Conclusions:** The original apparatus proposed by the authors determines a mesializing movement of the jaw through a different mechanism to traditional MADs and presents the great advantage of a digital and CAD–CAD workflow that can be developed directly by the clinicians in the practice.

## 1. Introduction

Respiratory sleep disorders occupy a prominent role in the landscape of so-called “emerging” diseases as they are closely linked to various systemic disorders, significantly affecting the general health condition of patients [1]. Sleep respiratory disorders are alterations in ventilatory and/or breathing mechanics of complex and often multidisciplinary origin that result in alteration of physiological sleep. The most common cause of physiological sleep alteration is obstructive sleep apnea (OSAS) [2]. During sleep, due to the position assumed by the body and as a result of muscle relaxation of the bundles that keep the airways pervious, there is a physiological reduction in the caliber of the first airways themselves. Under these conditions, airflow may be reduced by more than 50 percent (hypopneas) or stop for a few seconds (apneas). OSAS pathology affects subjects of all ages; in the pediatric population, the incidence varies between 1.2% and 5.7% [3], while in the adult population, it affects about 9% of females and 27% of males [4]. Night snoring is always associated with this pathology, although not all habitual snorers suffer from sleep apnea. OSAS has a significant impact on the patient’s overall neurocognitive function, often causing episodes of memory loss, alterations in personality, inability to concentrate, and reduced attention.

Appropriate and tempestive treatment significantly improves the patient’s clinical condition. The treatment of OSAS involves first of all the correction of predisposing factors such as risky lifestyles and overweight, and then, depending on the severity of the clinical picture, various therapeutic strategies such as the use of intraoral devices and continuous positive airway pressure (CPAP) up to surgical corrections; the therapeutic objective is always to widen the upper airway and reduce collapsibility. Non-surgical treatment of obstructive sleep apnea syndrome is based on the use of the continuous positive airway pressure apparatus and mandibular advancement device (MAD) [5]. Ventilatory therapy with CPAP allows significant improvement of nocturnal respiratory activity even in patients with severe OSAS; however, considering that it is an invasive device, it is not well tolerated by a high percentage of patients, with the consequence that in the medium and long terms, several patients drop out of therapy [6]. Despite its clinical efficacy, poor adherence to CPAP treatment remains a significant problem to date in the treatment of obstructive sleep apnea, especially in its more severe forms, and there is little evidence on how to improve it. However, the recent commercialization of increasingly high-performance and less invasive CPAP devices has not significantly improved patient compliance with this type of equipment, which is perceived as uncomfortable. Several studies have evaluated adherence to CPAP, which was found to be highly variable, ranging from 30% to 80%. Several studies have described factors associated with the abandonment of CPAP therapy. The Sleep Apnoea Cardiovascular Endpoints Study showed that the use of CPAP therapy was evidently decreased at 1 year in patients with moderate to severe OSAS associated with cardiovascular disease. The authors reported that adherence to CPAP and side effects of therapy at 1 month (dry mouth, nasal symptoms, eye problems, claustrophobia, hearing problems, facial pain, mask-related skin irritation, mask wear, and leakage) were predictors of a decreased rate of CPAP therapy use. Symptoms of rhinitis, dry/congested nose, and dry mouth or throat are more common in elderly patients and in those treated with dehydrating drugs or with previous nasal symptoms or surgery.

Mandibular advancing devices (MADs), on the other hand, are removable intraoral devices to be used during sleep that, thanks to the modification of the mandibular spatial position, increase airway patency, thus reducing the resistance to the transit of airflow in the nasopharyngeal tract [7]. Mandibular advancement devices are an alternative to CPAP for patients with mild or moderate obstructive sleep apnea/hypopnea syndrome; in cases where the patient refuses CPAP treatment, they can also be used for severe OSAS [8,9]. There are three different types of intraoral devices for the treatment of OSAS: palatal lifting devices, tongue retainer devices (TRDs), and mandibular advancement devices. Palatal lifting devices are no longer used in the clinical setting, while TRDs are only used in edentulous patients. Mandibular advancement devices can be standard boil-and-bite devices or customized devices. Today, it is preferred to use customized adjustable devices, which allow the creation of less bulky devices with a high retention, resulting in a more comfortable fit for the patient. Such devices allow, among other things, better lateral mandibular movement and progressive mandibular mesialization, thus reducing the risk of Temporo Mandibular Joint (TMJ) disorders. The use of MADs results in an increase in the subject’s respiratory capacity, an increase in blood oxygenation levels, and consequently an improvement in sleep quality. In traditional mandibular advancement devices, the modification of the spatial position of the mandible is achieved through the interlocking of resin valleys, screw systems, telescopic arm systems, etc.; however, all of these mandibular propulsion systems involve significant bulky equipment within the oral cavity. The objective of the present study is to propose a digital workflow for the fabrication through a CAD–CAM method of a mandibular advancement device that is based on the attraction of rare-earth magnets embedded in the thickness of two thermally molded PET-G templates.

## 2. Materials and Methods

**Figure 1:**
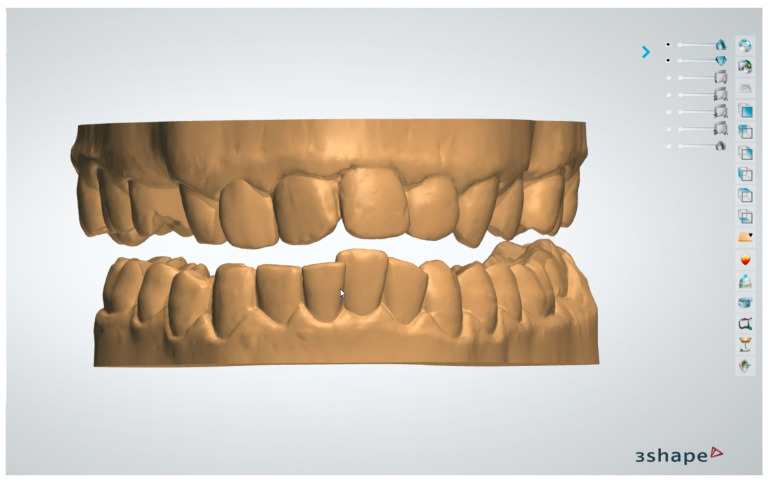
Digital models.

The original workflow proposed in the present study first of all involved a digital survey of both dental arches and recording occlusal relationships (Figure 1) using 3Shape TRIOS 4 intraoral scanner (3Shape A/S Company, Copenhagen K, Denmark).

**Figure 2:**
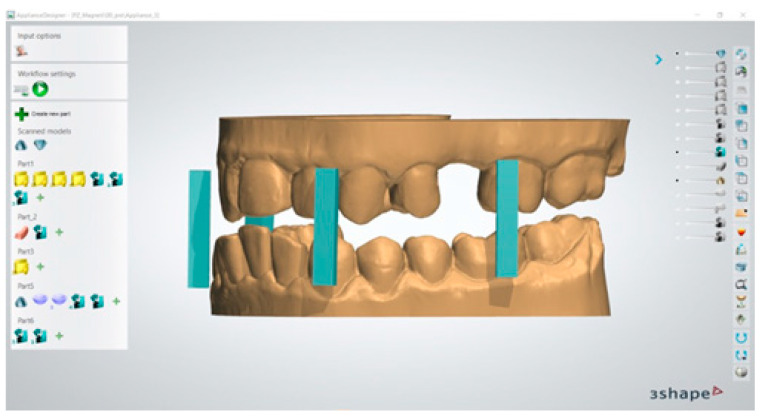
Magnetic digital model positioning.

Virtual copies of the magnetic device were imported within 3Shape device application software 3Shape Ortho System 2022 (3Shape, Copenhagen K, Denmark)) and positioned at the level of the molar and canine regions, as programmed in the device design phase (Figure 2); this was performed in order to achieve an effective magnetic attraction to promote the desired mandibular advancement position.

**Figure 3:**
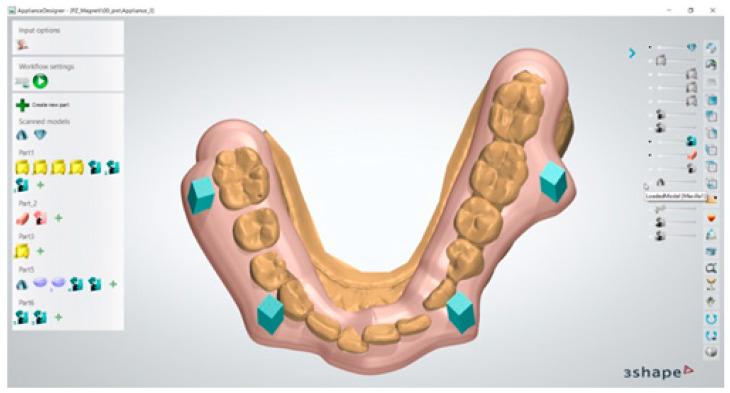
Digital transfer template with housing for magnetic segments.

Once the correct positioning of the magnetic segments was defined, a transfer template was digitally developed and conformed to provide a specific slot for the magnets at the level of the canines and first molars (Figure 3).

**Figure 4:**
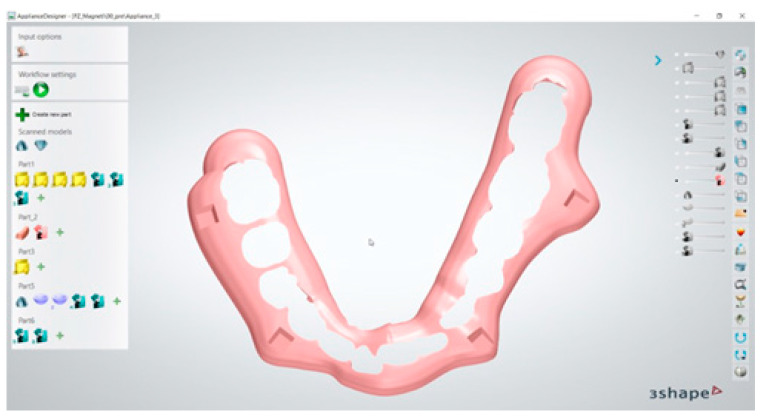
Three-dimensional model of transfer template.

The transfer template was created and then digitally extrapolated from the model while retaining in itself the information regarding the dental elements and magnet housing (Figure 4).

**Figure 5:**
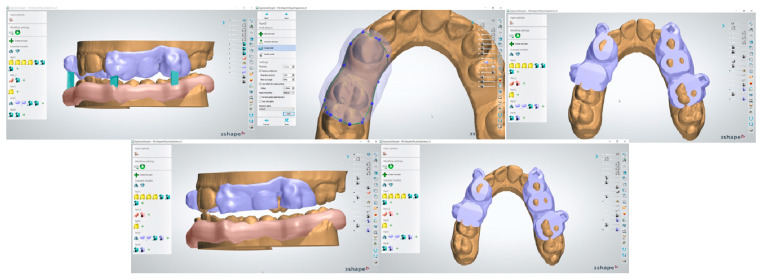
Digital verification of the correct relationship of devices.

After completing the same procedure for both dental arches, the correct relationship of the two devices was verified through 3Shape software 3Shape Ortho System 2022 (3Shape, Copenhagen K, Denmark) (Figure 5) before digitally printing the guide templates.

**Figure 6:**
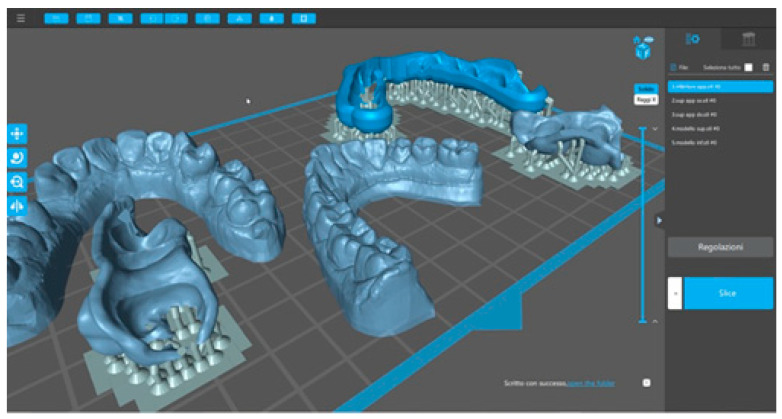
Three-dimensional printing.

The digitally developed device was three-dimensionally printed (Figure 6) using special certified resin (Impression Tray Resin LC, Henry Schein Inc., Melville, NY, USA).

**Figure 7:**
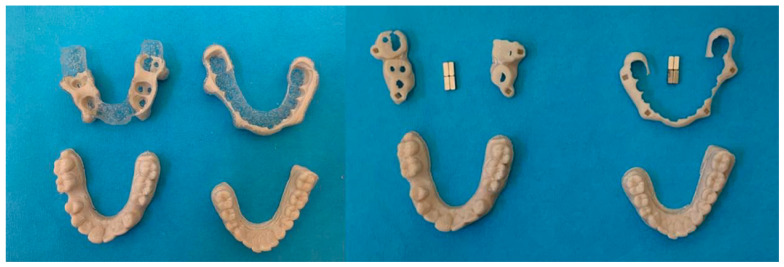
Assembly of the different components of the device.

The digital models, the basic structure of the mandibular advancement device with the housing for the magnets, and the magnets themselves were then assembled together in order to compose the final model of the device (Figure 7). The rare-earth magnets used were of the Magnet Cuboid YXG28H type (HKCM Engineering Eckernförde, Germany) and have a cuboid shape with dimensions of 3 × 3 × 8 mm (length–width–height) and a holding force of 4.22 Newtons.

**Figure 8:**
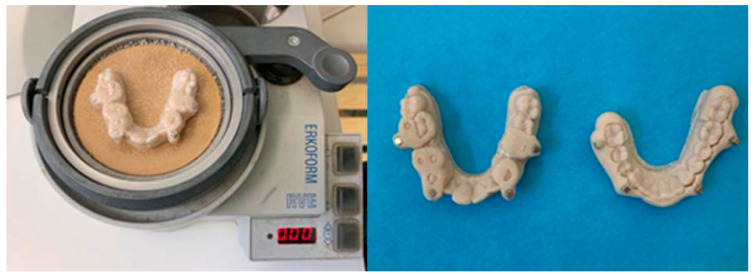
Device thermoforming.

The final composition of the device was obtained by thermoforming process using a sheet of thermoplastic material ERKODUR-S 1.0 mm and diam. 120.0 mm (Erkodent Erich Kopp GmbH Pfalzgrafenweiler, Germany with a thickness of 1 mm (Figure 8).

**Figure 9:**
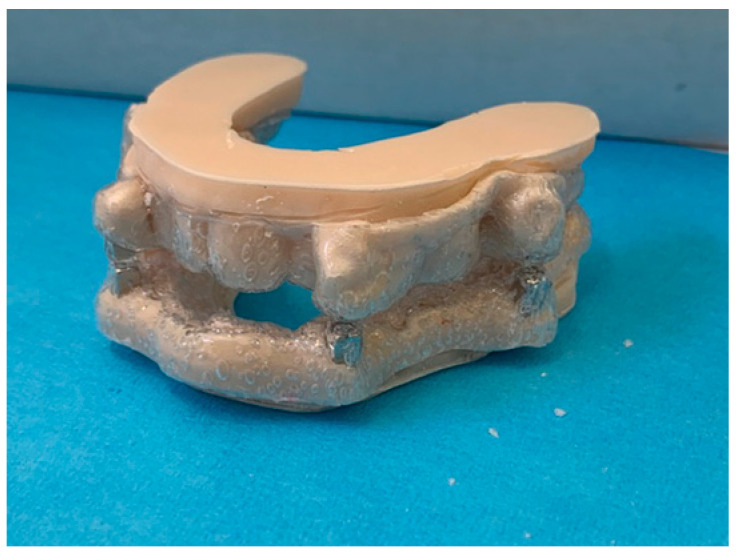
MAMA appliance.

Through digital workflow and by means of CAD–CAM technology, the original mandibular advancement device was thus fabricated (Figure 9).

The device was patented on 03-04-2023 at the Italian Ministry of Economic Development (concession no. 1020210007646), and the owner of the intellectual property is the University of Messina.

## 3. Results

The original digital workflow proposed by the authors allows the development of a comfortable and highly wear-resistant device that could be used for the treatment of mild and moderate OSAS. The innovative feature of the device proposed by the authors is that the mandibular propulsion induced by the attraction of the magnetic jigs inserted into the thickness of the masks could not be affected by the patient’s mandibular posture during sleep; the digital and CAD–CAD workflows proposed in this paper also present the great advantage that they can be developed directly by the dentist in the practice.

## 4. Discussion

The original device designed by the authors consists of two thermally molded PETG (copolymer of the polyester family, a variant of polyethylene terephthalate) templates, one for the maxillary arch and one for the mandibular arch, into the thickness of which cuboid-shaped rare-earth magnets of 3 × 3 × 8 mm (length–width–height) dimensions with a holding force of 4.22 Newtons were inserted. The force of attraction between the two magnets inserted into the plates caused the mandibular template to move closer to the maxillary template, resulting in passive mandibular advancement. The fabrication of a PET-G device compared with more traditional resin devices offers several clinical advantages related to the innovative characteristics of this [10,11,12] polymer. In fact, the most commonly used mandibular advancement devices to date are made of acrylic resin; however, these materials require rather substantial thicknesses [13] to ensure adequate mechanical characteristics. This implies that acrylic resin devices, on average, have volumes that result in significant bulk within the patient’s oral cavity and have a considerable impact in terms of comfort and esthetics [14]. PETG, on the other hand, is a particularly durable and remarkably flexible material with high fracture resistance [15,16]: shrinkage is quite low, consequently, the tendency to develop deformations is practically minimal [17,18]. This makes it possible to make mandibular advancement devices with very small thicknesses, which are therefore particularly comfortable for the patient; this will allow a significant reduction in the sense of bulk within the oral cavity during sleep, improving patient compliance in using the device. Despite the reduction in thickness, the device is still robust, resistant to deformation, and, therefore, durable. PETG is also extremely resistant to the action of chemicals, acids, and alkalis [19]; it is an ideal material for obtaining waterproof and durable models in wet environments such as the oral cavity [20]. PETG is a semitransparent material; this characteristic also makes it possible to obtain particularly esthetic devices with a high degree of strength, thus maintaining their esthetic characteristics over a long period of time without causing dulling and blemishing of the device [21,22]. An additional innovative feature of the device proposed by the authors is that the mandibular propulsion induced by the attraction of the magnetic jigs inserted into the thickness of the templates could be not affected by the patient’s mandibular posture. Therefore, if during sleep the OSAS patient tends to assume an open-mouth position, which could reduce the clinical effectiveness of conventional MAD devices, the mandibular advancement induced instead by magnetic attraction would not be affected in any way. Mandibular propulsion produced by the attraction of rare-earth magnets was unaffected by the patient’s mandibular posture, and it could be clinically more effective and significantly more prolonged during sleep time at night [23]. Some studies in the literature have shown that the forces generated by magnetic systems can stimulate bony neoapposition and thus result in remodeling at the level of the jaw bones [24], which could facilitate the correction of altered skeletal patterns that are very common in patients affected by OSAS. To date, we have not yet carried out a clinical trial demonstrating the efficacy of the original device patented; the aim of this paper is to explain in detail the different processing steps through which the device is made. Further studies are needed to evaluate the clinical efficacy of magnetic attraction advancement devices and their use in different degrees of obstructive sleep apnea syndrome.

## 5. Conclusions

Based on the results achieved in our study, the following can be stated:

- The digital workflow proposed in this study appears to be effective and efficient for the design of a magnetic advancement device;
- CAD–CAM technology allows us to make an individualized device for each patient with a high degree of precision;
- The original workflow proposed by the authors allows clinicians to develop the apparatus directly in the practice.

## 6. Patents

The device presented in this paper was patented on 03-04-2023 at the Ministry of Economic Development (concession no. 1020210007646).

## Data Availability

The original contributions presented in the study are included in the article, further inquiries can be directed to the corresponding author.

## References

[1] Sateia M.J. (2014). International classification of sleep disorders-third edition: Highlights and modification. Chest.

[2] Maspero C., Giannini L., Galbiati G., Rosso G., Farronato G. (2015). Obstructive sleep apnea syndrome: A literature review. Minerva Stomatol..

[3] Young T., Evans L., Finn L., Palta M. (1997). Estimation of the clinically diagnosed proportion of sleep apnea syndrome in middle-aged men and women. Sleep.

[4] Marcus C.L., Brooks L.J., Draper K.A. (2012). Diagnosis and management of childhood obstructive sleep apnea syndrome. Pediatrics.

[5] Xu T., You D., Chen X. (2018). Non-surgical treatment of obstructive sleep apnea syndrome. Eur. Arch. Otorhinolaryngol..

[6] Loução-de-Amorim I., Bentes C., Peralta A.R. (2019). Men and women with chronic insomnia disorder and OSAS: Different responses to CPAP. Sleep. Sci..

[7] Serra Torres S., Bellott Arcis C., Montiel Company J.L., Marco Algarra J., Almerich Silla J.M. (2016). Effectiveness of mandibular advancement appliances in treating obstructive sleep apnea syndrome: A systematic review. Laryngoscope.

[8] Kapur V., Auckley D.H., Chowdhuri S., Kuhlmann D., Mehra R., Ramar K., Harrod C.G. (2017). Clinical Practice Guideline for Diagnostic Testing for Adult Obstructive Sleep Apnea: An American Academy of Sleep Medicine Clinical Practice Guideline. J. Clin. Sleep. Med..

[9] Ping L., Xiao-Hui N., Hua L., Ning Z., Yan-Feng G., Fen P. (2020). Continuous positive airway pressure versus mandibular advancement device in the treatment of obstructive sleep apnea: A systematic review and meta-analysis. Sleep. Med..

[10] Jindal P., Juneja M., Siena F., Bajaj D., Breedon P. (2019). Mechanical and geometric properties of thermoformed and 3D printed clear dental aligners. Am. J. Orthod. Dentofac. Orthop..

[11] Casavola C., Pappalettera G., Pappalettere C., Patronelli M., Renna G., Laurenziello M., Ciavarella D. (2022). A full-field DIC analysis of the mechanical-deformation behavior of polyethylene terephthalate glycol (PET-G) aligners. J. Mech. Behav. Biomed. Mater..

[12] Iliadi A., Koletsi D., Eliades T. (2019). Forces and moments generated by aligner-type appliances for orthodontic tooth movement: A systematic review and meta-analysis. Orthod. Craniofac Res..

[13] Shirey N., Mendonca G., Groth C., Berman H. (2023). Comparison of mechanical properties of 3-dimensional printed and thermoformed orthodontic aligners. Am. J. Orthod. Dentofac. Orthop..

[14] Zinelis S., Panayi N., Polychronis G., Papageorgiou S., Eliades T. (2022). Comparative analysis of mechanical properties of orthodontic aligners produced by different contemporary 3D printers. Orthod. Craniofac Res..

[15] Lombardo L., Martines E., Mazzanti V., Arreghini A., Mollica F., Siciliani G. (2017). Stress relaxation properties of four orthodontic aligner materials: A 24-hour in vitro study. Angle Orthod..

[16] Skaik A., Wei X., Abusamak I., Iddi I. (2019). Effects of time and clear aligner removal frequency on the force delivered by different polyethylene terephthalate glycol-modified materials determined with thin-film pressure sensors. Am. J. Orthod. Dentofac. Orthop..

[17] Ahn H., Lee S., Yu H., Park J., Kim K., Kim S. (2021). Force Distribution of a Novel Core-Reinforced Multilayered Mandibular Advancement Device. Sensors.

[18] Elkholy F., Schmidt S., Schmidt F., Amirkhani M., Lapatki B. (2023). Force decay of polyethylene terephthalate glycol aligner materials during simulation of typical clinical loading/unloading scenarios. J. Orofac. Orthop..

[19] Nicita F., D’Amico C., Filardi V., Spadaro D., Aquilio E., Mancini M., Fiorillo L. (2024). Chemical-Physical Characterization of PET-G-Based Material for Orthodontic Use: Preliminary Evaluation of micro-Raman Analysis. Eur. J. Dent..

[20] Ihssen B., Willmann J., Nimer A., Drescher D. (2019). Effect of in vitro aging by water immersion and thermocycling on the mechanical properties of PETG aligner material. J. Orofac. Orthop..

[21] Bernard G., Rompré P., Tavares J.R., Montpetit A. (2020). Colorimetric and spectrophotometric measurements of orthodontic thermoplastic aligners exposed to various staining sources and cleaning methods. Head. Face Med..

[22] Porojan L., Vasiliu R., Porojan S., Bîrdeanu M. (2020). Surface Quality Evaluation of Removable Thermoplastic Dental Appliances Related to Staining Beverages and Cleaning Agents. Polymers.

[23] Vardimon A.D., Graber T.M., Voss L.R., Muller T.P. (1990). Functional orthopedic magnetic appliance (FOMA) III--modus operandi. Am. J. Orthod. Dentofac. Orthop..

[24] Nalabothu P., Verna C., Steineck M., Mueller A.A., Dalstra M. (2020). The biomechanical evaluation of magnetic forces to drive osteogenesis in newborn’s with cleft lip and palate. J. Mater. Sci. Mater. Med..

